# Falls efficacy: The self-efficacy concept for falls prevention and management

**DOI:** 10.3389/fpsyg.2022.1011285

**Published:** 2022-11-09

**Authors:** Shawn Leng-Hsien Soh

**Affiliations:** ^1^Health and Social Sciences Cluster, Singapore Institute of Technology, Singapore, Singapore; ^2^Centre for Health, Activity and Rehabilitation Research, Queen Margaret University, Musselburgh, United Kingdom

**Keywords:** falls efficacy, self-efficacy, older people, falls prevention, falls management, balance confidence, fear of falling, balance recovery confidence

## Introduction

Falls efficacy has been keenly studied in older people since the introduction of the Falls Efficacy Scale. The seminal paper “Falls efficacy as a measure of fear of falling” has received over 2,500 citations since 1990 (Tinetti et al., 1990). The presence of newer versions of falls efficacy-related scales, either modified from the ancestral version or constructed afresh, suggests that many researchers and clinicians are interested in investigating the meaningful impact of falls efficacy (Soh et al., 2021a). Ultimately, endeavors aiming to empower older people to prevent and manage falls need a clear understanding of falls efficacy. Some articles featured in the Frontiers have demonstrated these efforts, such as presenting the mediating role of falls efficacy between fatigue and falls risk (He et al., 2022) and the role of a falls risk-reduction program on falls efficacy (Cho et al., 2014). Given the advances in research on falls efficacy, have we adequately understood this self-efficacy concept and have the most appropriate measure applied for the construct of interest?

Literature has reported several challenges in understanding falls efficacy since the 2000s. Two systematic reviews reported significant difficulties in deciphering whether the measures of falls efficacy were measuring falls efficacy, balance confidence or fear of falling (Jorstad et al., 2005; Moore and Ellis, 2008). Various researchers have attempted to clarify the falls efficacy concept. Hadjistavropoulos et al. (2011) presented key research findings to advocate that falls efficacy and balance confidence are *equivalent and interchangeable*. However, Hughes et al. (2015) drew on the theoretical origins of falls efficacy, balance confidence and outcome expectancy to recommend that researchers clarify the different constructs. Recently, Soh et al. (2021b) posited that falls efficacy and balance confidence are dissimilar and that falls efficacy encompasses four domains surrounding falls (i.e., pre-fall, near-fall, fall-landing, and completed-fall). Elucidating falls efficacy as a broader set of perceived capabilities would advocate a complete approach to helping older people overcome falls and falling.

This commentary aims to update the understanding of falls efficacy by revisiting Bandura's self-efficacy theory and then offering a contemporary interpretation. The commentary highlights some selected measures to suggest that appropriate measures should be applied in research surrounding falls efficacy.

## Theoretical origin

Falls efficacy is rooted in Bandura's self-efficacy theory (Tinetti et al., 1990). Self-efficacy is the belief in personal capabilities to organize and execute the sources of action required to manage prospective situations (Bandura, 1997). As a cognitive mechanism, self-efficacy mediates between thoughts/emotions and actions (Bandura, 1986). The theory of self-efficacy postulates that perceived capability (i.e., level of confidence), other than the person's true capability, can influence behavior (Bandura, 1997). It is noteworthy to recognize that the efficacy belief system is a differentiated set of self-beliefs linked to distinct realms of functioning (Bandura, 2006). A fuller understanding of falls efficacy will provide more strategies to help individuals deal with falls and overcome concerning issues (i.e., fear of falling and fear-related activities-avoidance behavior).

## Evolution of the concept

Falls efficacy was first introduced to rationalize a novel scale, the Falls Efficacy Scale, to measure fear of falling (Tinetti et al., 1990). There were a few reasons. First, the theoretical framework of the scale would be grounded in Bandura's self-efficacy concept (Bandura, 1986). Second, fear could be purportedly determined through a continuous scale. Third, fear would be dissociated from psychiatric connotations. However, a follow-up study by the developers reported that the Falls Efficacy Scale should remain an efficacy measure rather than a fear measure because older people who were reportedly efficacious also feared falling (Tinetti et al., 1994).

Falls efficacy was then associated with the perceived ability to perform various activities steadily (balance confidence) (Hadjistavropoulos et al., 2011). Falls efficacy (i.e., balance confidence) was shown to improve by interventions, such as Tai Chi (Chewning et al., 2020), but not for those that improve performances of reactive balance (Kurz et al., 2016) or safe falling (Arkkukangas et al., 2020). These interventions might have improved other perceived self-efficacy to overcome falls, such as balance recovery confidence and safe-landing confidence. However, their effectiveness remains inconclusive due to the measures' limitations. Balance confidence measures aim to determine the perceived ability to perform activities steadily rather than to recover the balance from perturbations or fall safely on the floor (Soh et al., 2021a).

Falls efficacy has recently been posited to encompass four different types of self-efficacy surrounding falls: balance confidence, balance recovery confidence, safe-landing confidence, and post-fall recovery confidence (Soh et al., 2021b). Balance confidence relates to the perceived self-efficacy of performing activities without losing balance (Powell and Myers, 1995). Balance recovery confidence refers to the perceived ability to recover balance and arrest a fall in response to perturbations that can occur in everyday activities (Soh et al., 2022b). Balance recovery confidence differs from balance confidence, given that balance confidence focuses on the perceived capability to perform activities steadily, such as climbing up or down stairs. In contrast, balance recovery confidence refers to the perceived reactive balance recovery reactions, such as grabbing onto a handrail or taking a few steps to recover balance in response to a trip or a slip. Safe-landing confidence refers to the self-efficacy of falling safely onto the ground by minimizing landing injuries (Moon and Sosnoff, 2017). Fall recovery confidence refers to the self-efficacy to recover from falls, such as getting up or getting help (Hofmeyer et al., 2002). The concept was substantiated based on a review paper investigating the methodological quality of content developed for different falls efficacy-related measurement instruments (Soh et al., 2021a).

## Measurement instruments of falls efficacy

Measures of falls efficacy can be categorized into two broad types: single-domain and multi-domain. Single-domain measures that focus on one domain of falls efficacy rely on a well-defined conceptual analysis of the specific domain (De Vet et al., 2011). In contrast, multi-domain measures that encompass more than one domain of falls efficacy aim to reveal a general sense of personal efficacy to produce certain attainment (Bandura, 2006), that is, to overcome falls threat. However, a significant number of existing falls efficacy measures lack high quality evidence in their content development and validity based on a systematic review (Soh et al., 2021a) that applied the COSMIN methodology (COSMIN., 2021). Nevertheless, some of the measures commonly used in falls practice (APTA Geriatrics., 2021) that potentially fit into the current understanding of falls efficacy are presented in Table 1.

**Table 1:** A list of potential measurement instruments for balance confidence, balance recovery confidence, fall recovery confidence, and falls efficacy based on a systematic review conducted by Soh et al. (2021a).

**Single domain measures**			
**Name**	**Targeted construct^a^**	**About**	**Measurement properties based on COSMIN quality rating^b^**
Falls Efficacy Scale (Tinetti et al., 1990)	Balance confidence	A 10-item scale measures the perceived ability to perform various activities of daily living without falling. The scale uses a 10-point rating scale from 1 (very confident) to 10 (not confident at all) to determine a total score between 10 and 100.	Content development: Insufficient (absence of target population involvement). Content validity: Inconsistent with moderate quality evidence. Structural validity: Sufficient with high quality evidence.
Activities-specific Balance Confidence Scale (Powell and Myers, 1995)	Balance confidence	A 16-item scale measures the perceived ability to perform various activities of daily living without losing balance or becoming unsteady. The scale uses an 11-point rating scale from 0% (no confidence) to 100% (complete confidence) to determine a percentage of self-confidence in balance performance.	Content development: Indeterminate (unclear methods). Content validity: Indeterminate (unclear methods). Structural validity: Sufficient with high quality evidence.
Balance Recovery Confidence Scale (Soh et al., 2022b)	Balance recovery confidence	A 19-item scale measures the perceived ability to arrest falls in response to a trip, a slip, or a loss of balance presented in different scenarios. The scale uses an 11-point rating scale from 0 (cannot do at all) to 10 (highly certain can do) to determine self-confidence in reactive balance recovery performance.	Content development: Sufficient with high quality evidence. Content validity: Sufficient with moderate quality evidence. Structural validity: Sufficient with high quality evidence.
Difficulty Scale on Performance of Rising from the Floor (Hofmeyer et al., 2002)	Fall recovery confidence	A 7-item scale measures the perceived ability to get up from the floor. The scale uses a 4-point rating scale from 1 (no difficulty) to 4 (unable) to determine a total score between 7 and 28.	Content development: Insufficient (absence of target population involvement). Content validity: Insufficient (absence of target population involvement). Structural validity: Indeterminate (not investigated).
**Multi-domain measures**			
**Name**	**Targeted construct**	**About**	**Measurement properties based on COSMIN quality rating**
Perceived Ability to Prevent and Manage Fall Risks Scale (Yoshikawa and Smith, 2019)	Falls efficacy	A 6-item scale measures the perceived ability to prevent and manage falls. The scale uses a 5-point rating scale from 1 (excellent) to 5 (poor) to determine a score between 6 and 30. Assessing six different domains: “Steadiness on your feet,” “Balance while walking,” “Ability to walk in your home,” “Ability to walk outdoors,” “Ability to prevent falls,” and “Ability to find a way to get up if you fall.”	Content development: Insufficient (absence of target population involvement). Content validity: Insufficient (absence of target population involvement). Structural validity: Sufficient with high quality evidence.

a Measures for constructs, such as balance recovery confidence and post-fall recovery confidence, have not been validated rigorously. A measurement instrument for balance recovery confidence was recently developed in 2022 (Soh et al., 2022b). The difficulty scale on performance of rising from the floor (Hofmeyer et al., 2002) and the perceived ability to prevent and manage fall risks scale (Yoshikawa and Smith, 2019) were created as part of an intervention program. These measures should be applied cautiously.

b Four ratings (sufficient, insufficient, inconsistent, and indeterminate) are used to present the measurement property with the quality of the evidence (high, moderate, low, very low evidence) based on COSMIN methodology (Prinsen et al., 2018).

## Discussion

The concept of falls efficacy has evolved. Translating new insights of falls efficacy into practice promotes new and novel approaches to help older people overcome the threat of falls. In highlighting the different perceived self-efficacies, researchers and clinicians should select the most appropriate measures when studying the impact of different interventions on the construct of interest (McKenna et al., 2019). For example, exercise interventions, such as Pilates (Roller et al., 2018; Aibar-Almazan et al., 2019), Tai Chi (Okuyan and Bilgili, 2017; Chewning et al., 2020), Otago exercises (Johnson et al., 2021) and Fall Management Exercise programme (FaME) (Iliffe et al., 2015), being more holistic, could consider using multi-domain measures. Skill training interventions, such as Chinese martial arts training (Ma et al., 2019), perturbation training (Kurz et al., 2016; Lurie et al., 2020), Judo4Balance (Arkkukangas et al., 2020), and backward chain training (Leonhardt et al., 2020), being more focused strategies, could benefit from using single-domain measures for the targeted constructs.

Past research has primarily focused on balance confidence as an outcome (Soh et al., 2022a). A review of falls efficacy-related studies found that 90% employed either the Falls Efficacy Scale or the Activities-specific Balance Confidence Scale. Through these measures, the effect on balance recovery confidence, safe-landing confidence, and fall recovery confidence remains unclear. Single-domain measures should be appropriately used. However, completing several self-reported questionnaires can be burdensome. Multi-domain measures, such as the Perceived Ability to Prevent and Manage Fall Risks Scale (Yoshikawa and Smith, 2019), could be considered to obtain a general sense of perceived self-efficacy in preventing and managing falls. It is, however, imperative that these measures are used cautiously. Unlike the FES and ABC scale, these measures have not been rigorously validated. There is an urgent need for validation studies to critically evaluate falls efficacy-related measures and present their measurement properties.

Researchers and clinicians who want to measure fear of falling should use measures such as the Falls Efficacy Scale-International (FES-I) (Yardley et al., 2005), short FES-I (Kempen et al., 2008), Fear of Falling Questionnaire (Bower et al., 2015), and Fear of Falling Avoidance Behavior Questionnaire (Landers et al., 2011). Unlike self-efficacy instruments, these fear of falling measures were constructed to determine concerns about falling. These measures potentially capture multiple expectancies, such as perceived consequences arising from a fall, perceptual control, and their judgement of capability to act in the given scenarios (Lach, 2006). Falls efficacy measures should be complementarily used with the fear of falling measures. A fuller picture of the different psychological factors is essential in predicting falls and determining performance (Hadjistavropoulos and Delbaere, 2021). Thoughtful employment of an enduring self-efficacy concept in falls research and clinical work will help advance novel interventions to address the person's self-development and behavioral adaptation and changes.

In conclusion, falls efficacy can be viewed as a perceived ability to prevent and manage falls. Embracing this interpretation provides a broader paradigm toward helping older adults be more resilient against falls. Applying appropriate measures for the perceived capability in preventing and managing falls is imperative to clarify the targeted construct.

## Author contributions

The author confirms being the sole contributor of this work and has approved it for publication.

## Conflict of interest

The author declares that the research was conducted in the absence of any commercial or financial relationships that could be construed as a potential conflict of interest.

## Publisher's note

All claims expressed in this article are solely those of the authors and do not necessarily represent those of their affiliated organizations, or those of the publisher, the editors and the reviewers. Any product that may be evaluated in this article, or claim that may be made by its manufacturer, is not guaranteed or endorsed by the publisher.

## Author disclaimer

The opinions given in this article are those of the author and do not necessarily represent the official position of Queen Margaret University and Singapore Institute of Technology.
